# Evaluation of Occupational Health and Safety Management of Listed Companies in China’s Energy Industry Based on the Combined Weight-Cloud Model: From the Perspective of FPE Information Disclosure

**DOI:** 10.3390/ijerph19148313

**Published:** 2022-07-07

**Authors:** Yujie Wang, Hong Chen, Ruyin Long, Shiyan Jiang, Bei Liu

**Affiliations:** 1 School of Economics and Management, Taiyuan University of Technology, Taiyuan 030600, China; wangyujiesx@163.com; 2 School of Business, Jiangnan University, Wuxi 214122, China; longruyin@163.com; 3 Institute of National Security and Green Development, Jiangnan University, Wuxi 214122, China; 4 School of Economics and Management, China University of Mining and Technology, Xuzhou 221116, China; jsy19951007@163.com (S.J.); liubie@163.com (B.L.)

**Keywords:** listed companies in the energy industry, occupational health and safety management, cloud model, FPE information disclosure

## Abstract

Protecting labor safety and health and actively carrying out occupational safety and health management (OSHM) is a common need worldwide, and it is also one of the important efforts of Chinese enterprises under the background of promoting the implementation of the Healthy China strategy. Based on in-depth thinking on the current stage of OHSM, this study incorporated “management framework, management process, management effectiveness” (FPE) into an integrated framework and constructed an FPE evaluation system for enterprise OHSM. This study innovatively collected and refined FPE information from the perspective of information disclosure and used the combined weight cloud model to evaluate the occupational health and safety management level (OHSML) of 69 listed companies in China’s energy industry from 2009–2019. The results showed the following. (1) The OHSML of most listed companies in China’s energy industry was still at a low-end level. Among the companies that have issued relevant information reports, only 5.58% (S = 30) of the sample companies’ OHSML were at an acceptable level (Level IV) or declarable level (Level V). The OHSML comprehensive evaluation level of 92.56% (S = 498) of the sample companies was between the transitional level (Level III) and the improved level (Level II). (2) During 2009–2019, although the annual OHSML of listed companies in China’s energy industry showed an upward trend, the growth rate was low, and even the OHSML of some listed companies in the energy industry showed the characteristics of reduced fluctuations. (3) From the perspective of the PFT three-dimensional subsystem level of OHSM, the evaluation level of the governance framework subsystem was the highest, whereas the evaluation level of the management process subsystem and the management effectiveness subsystem were relatively low. Finally, according to the relevant results, some suggestions were proposed to improve the OHSML of listed companies in China’s energy industry. These findings can provide guidance for companies to improve their OSHM performance.

## 1. Introduction

The problem of occupational health and safety (OHS) has become a significant challenge that all countries face and urgently need to solve in modern industrial construction. According to estimates by the International Labor Organization, there are 250 million casualties and 2.34 million deaths in the world every year, among which 2.02 million died of occupational diseases, and these numbers are increasing [1]. The annual economic loss caused by inadequate OHS measures accounts for approximately 4% of the global annual GDP, about 3.3 trillion US dollars [2]. In addition, with the continuous intensification of global economic competition and changes in production and lifestyles, occupational hazards have also shown new characteristics. Traditional occupational diseases (such as pneumoconiosis) are still widespread, whereas new types of occupational diseases (such as chronic pain and mental occupational diseases now becoming recognized as occupational illnesses) are showing a sharp rise. In this context, to promote the construction of a healthy China and improve people’s living standards, China has issued a series of laws and regulations, such as the “Healthy China 2030” Planning Outline [3,4,5]. These clearly point out that “putting health in a strategic position as a priority for development”, “strengthening safety production supervision in key industries” and “strengthening the occupational disease reporting system” are of key importance. At the same time, these laws and regulations also put forward a series of specific goals, such as improving the occupational disease prevention and control systems, improving the ability of occupational disease monitoring, and protecting the health rights of workers. The energy industry, including coal, petroleum, and electric power, plays an important role in economic development. It is a basic industry for the development of China’s national economy, and it has always been an industry in China with a high incidence of safety accidents and occupational diseases. Therefore, enterprises in this industry have become the critical focus units for implementing the “Healthy China” strategy, and their occupational health and safety management (OHSM) status will have a significant impact on the effectiveness of the implementation of that strategy. In this context, fully clarifying the development status of OHSM of listed companies in the energy industry, mobilizing them to carry out OHSM actively, and guiding them to improve the weak links of OHSM have become important topics for China’s sustainable development.

Scholars have carried out relevant research from different perspectives regarding safety and health issues in the workplace. Existing research mainly focuses on OHSM models and methods, OHSM systems and standards, OSH risk assessment management, safety culture/atmosphere, mental health and quality of life, specific disease management, etc. [6]. Several studies have evaluated the OHSM status of the enterprise. For example, Lamontagne et al. (2004) evaluated 15 OSH projects for manufacturing jobs based on the adapted OSH project evaluation index [7]. Rajendran and Gambatese (2009) applied the concept of sustainable development to the OHSM of the construction industry, developed an OHSM rating system for the sustainable construction industry, and rated the OHSM status of the construction industry [8]. Yan et al. (2017) used Spearman’s correlation coefficient method to identify the critical factors of the health and safety environment (HSE) performance of oil companies based on historical data and used a dynamic fuzzy evaluation method to evaluate the HSE status of large oil companies [9]. Taylan et al. (2017) constructed a set of primary and secondary standards from two aspects of qualitative and quantitative evaluation and used fuzzy decision trees and TOPSIS methods to evaluate the OHS performance of 21 food manufacturing companies [10]. Tremblay and Badr (2018) developed a new OHS performance evaluation tool that is more suitable for SMEs and used forestry/pulp and paper SMEs as an example for evaluation and application [11]. However, these studies mostly used field surveys to study a few companies. The measurement of the occupational health and safety management level (OHSML) still heavily relies on consequence indicators such as the rate of industrial accidents, the rate of casualties, and the incidence of occupational diseases [12,13]. There is still a lack of research on systematically and comprehensively evaluating the OHSML status of enterprises from the perspective of management practice.

In terms of OHSM evaluation methods, most studies have adopted qualitative or semi-qualitative methods such as expert scoring, employee self-reports, questionnaire surveys, and fuzzy comprehensive evaluation methods to conduct research [14,15]. Although some methods have considered the uncertain factors of information in the evaluation process, they cannot fully and objectively evaluate the randomness. The cloud model is a modern mathematical method that specializes in studying complex uncertainty problems [16]. As this method can better present the randomness, fuzziness, and correlation of the variables [17], as well as realize the mapping and transformation between qualitative and quantitative uncertainties [18], it has been widely applied in robot state assessment [19], safety performance assessment [20], spatial suitability assessment [21], disaster risk assessment, and other fields [22]. There are relatively few studies on OHSM assessment of listed companies in the energy industry. On one hand, the number of listed companies in the energy industry is large, and the applicability of questionnaire surveys and other methods is poor. On the other hand, there are many indicators to measure the OHSM level of listed companies in the energy industry, including both quantitative and qualitative indicators, so it is difficult to conduct a comprehensive and objective assessment by expert scoring and other methods. The comprehensive evaluation method based on the cloud model can precisely realize the integrated evaluation of qualitative and quantitative indicators and can better describe the randomness and fuzziness of numerical values so as to achieve a more comprehensive and objective evaluation. Based on these factors, this study uses the cloud evaluation model to evaluate the OHSM level of listed companies in the energy industry.

Based on the in-depth thinking of OHSM practice, this study incorporated “management framework, management process, management effectiveness” (FPE) into an integrated framework and constructed an FPE evaluation system for enterprise OHSM. The study then innovatively collected and refined FPE information from the perspective of information disclosure. Furthermore, the combined weight-cloud model was used to evaluate the OHSML of listed companies in the energy industry in China. The objectives of this paper are to clarify the status and weaknesses of the OHSM of listed companies in the energy industry, to sort out the management benchmarking enterprises and key improvement indicators of various industries, and then to provide a reference for enterprise OHSM in the new era, with a view to providing reference for guiding companies to improve OHSML.

The innovations of this paper are as follows. (1) Based on in-depth thinking about the existing OHSM practice, the “management framework, management process, management effectiveness” (FPE) was incorporated into the integrated framework, and the FPE evaluation system of enterprise safety and health management was constructed. (2) Combined with the information disclosure measurement method, a qualitative index rating basis was constructed, and the FPE-related information was collected and refined innovatively from the perspective of information disclosure. (3) An enterprise OHSM evaluation model based on the combined weight-cloud model was constructed, and an integrated evaluation of qualitative and quantitative indicators was realized.

## 2. Methodology

### 2.1. Evaluation Framework for Enterprise OHSM

At present, the evaluation research on OHSM practices of enterprises at home and abroad has been mainly carried out from the lagging indicators, such as the rate of industrial accidents, the rate of casualties, and the incidence of occupational diseases [13,23]. Although some scholars incorporated management process indicators into the OSH management evaluation of enterprises for research, these studies mostly used field surveys to study a few enterprises. The measurement of OHSM mainly focused on safety education and training, emergency management, etc., and less attention was paid to leading indicators such as OSH department/personnel settings, hidden danger investigation and management activities, and mental health management [24,25]. Many studies have shown that leading indicators such as OSH education and training are more predictive of the future performance of enterprise OSH management than lagging indicators such as safety accident rate. It is an effective tool to measure the level of OHSM [26,27]. This study believes that the connotation of enterprise OHSM should continue to deepen, with the deepening of people’s systematic understanding of enterprise OSH. Enterprise OHSM should expand from only focusing on safety performance to the entire management system field. That is, the connotation of enterprise OHSM should be a complete interpretation of the three major elements of “management framework, management process, management effectiveness”. However, no matter from the perspective of theoretical research or enterprise practice, few scholars or organizations have systematically and comprehensively studied the OHSM status of enterprises from management’s perspective and incorporated the “framework -process-effectiveness” in the operation process of OHSM system into the integration framework. Therefore, based on the whole life cycle theory of management process, this study incorporated “management framework, management process, management effectiveness” (FPE) into the integrated framework and proposed an FPE evaluation framework for enterprise OHSM.

### 2.2. Evaluation Index System for Enterprise OHSM

Guided by the FPE evaluation framework of OHSM proposed in a previous paper, this study constructs a specific evaluation index system from three aspects of the management framework, management process, and management effectiveness. By referring to a large number of relevant literatures and relevant laws and regulations [9,28] combined with the actual situation of enterprise OHSM, and strictly following the design principles of scientific, systematic, comparable, operable, and other indicators, this study constructed an enterprise OHSM evaluation index system. The system was divided into four layers: target layer, subsystem layer, element layer, index layer (Table 1). The selection of indicators at each subsystem level is explained as follows:

(1) Management framework: A reasonable governance framework can determine the company’s OHSM vision, culture, strategy, system, etc., from the top-level design, which is the key to improving the company’s OHSM level and sustainable development. For example, Cornelissen et al. (2014) proposed that forward-looking safety system design could help create a healthier and safer working environment, thereby improving safety performance [29]. Molenaar et al. (2009) proposed that safety culture and its characteristics could be used as a measure of enterprise safety performance [30]. Mohammadfam et al. (2017) found through empirical research that the OHSM performance of companies certified by the OHSM system is significantly better than that of non-certified companies [31]. Therefore, this study believes that the enterprise OHSM evaluation system should cover the element of the management framework, and we have set up specific indicators such as C1~ institution system, C2~ management culture, C3~ management system, and C4~ terms and policies and other specific indicators to evaluate the top-level design of enterprise OHSM.

(2) Management process: Continuous attention to safety and health management in the production and operation process is essential to improving the company’s OHSM level and sustainable development capabilities. Some scholars have also evaluated and studied the OHSM status of enterprises from the management process level. For example, Fu et al. (2013) studied the relationship between education and training and OHS in enterprises, and the results showed that participatory training effectively improved the occupational health status of Chinese small and medium-sized enterprises [32]. Sorensen et al. (2014) put forward that OHSM should be evaluated from supporting organizational policies and practices (including accountability and training), incentives to support workplace health promotion and protection, comprehensive monitoring, and supervision through the integration of related research [33]. Pawłowska (2015) proposed that OSH performance can be measured by lagging indicators (outcome indicators such as occupational accident rate, number of sick days, number of occupational patients, etc.) and leading indicators (activity indicators such as education and training coverage, protective measures, and investigation of hidden dangers). Among them, leading indicators were often used in companies with higher performance levels [12]. Therefore, this study believes that the enterprise OHSM evaluation system should cover this element of the management process and set up specific indicators such as C5~project topics, C6~education and training, C7~monitoring and protection, C8~prevention and control, C9~disease management, and other specific indicators to evaluate the implementation process of enterprise OHSM.

(3) Management effectiveness: Management effectiveness is the most intuitive and vital manifestation of enterprise safety and health management. It can convey the management status of the company to investors, and it can also objectively reflect the level of the company’s OHSM. Governance effectiveness indicators mainly reflect the performance of enterprises in terms of safety accidents, occupational disease incidence, particular investment, and management impact. Many scholars introduced these indicators in their research to measure the OHSM status of enterprises. For example, Liu Suxia et al. (2014) measured the safety performance of small and medium-sized enterprises from safety consequence indicators such as the number of casualties, the number of accidents, the number of economic losses, and the number of occupational injuries [34]. Chen Chun (2010) constructed an indicator system based on the literature case method and evaluated the safety management performance of Chinese copper mining enterprises in terms of safety education and training, accidents, emergency management, etc. [35]. Therefore, this study believes that the enterprise OHSM evaluation system should cover the element of management effectiveness and set up C10~safety accident, C11~occupational disease, C12~continuous improvement, C13~management impact, and other specific indicators to evaluate the management effectiveness of enterprise OHSM.

### 2.3. Research Samples and Data Sources

Listed companies in the energy industry are typical representatives of outstanding companies in the energy industry, and their OHSM status will have a significant impact on the implementation of the “Healthy China” strategy. Based on this, this study selects listed companies in the energy industry as the research sample. The specific research objects are derived from the coal mining and washing industry, oil and gas extraction and processing industry, electricity and heat production and supply industry, gas heat production and supply, and water production and supply industry in the “Guidelines for Industry Classification of Listed Companies” issued by the China Securities Regulatory Commission. After sample screening (excluding ST and *ST companies; companies listed after 2009; and companies that have not disclosed social responsibility information), 538 observation samples of 69 listed companies in the energy industry from 2009 to 2019 were finally obtained.

The basic data of this research can be divided into two types: quantitative index data and qualitative index data. The specific data come from relevant information reports released by listed companies in the energy industry, such as social responsibility reports, sustainable development reports, employee safety and health reports, environment, society, and governance reports, etc. Among them, the quantitative index can be obtained or calculated through social responsibility reports, CSMAR database, the company’s official website, and other channels to obtain indicator data values, such as safety accident rate, the new incidence of occupational diseases, per capita safety and health investment, etc. However, qualitative indexes themselves are difficult to quantify, and need to be quantified in conjunction with expert scoring and information disclosure measurement methods. With reference to related literature [36,37], this study uses a score of 1 to 5 to quantify this type of index.

### 2.4. Comprehensive Evaluation Model Based on the Combined Weight-Cloud Model

**Figure 1:**
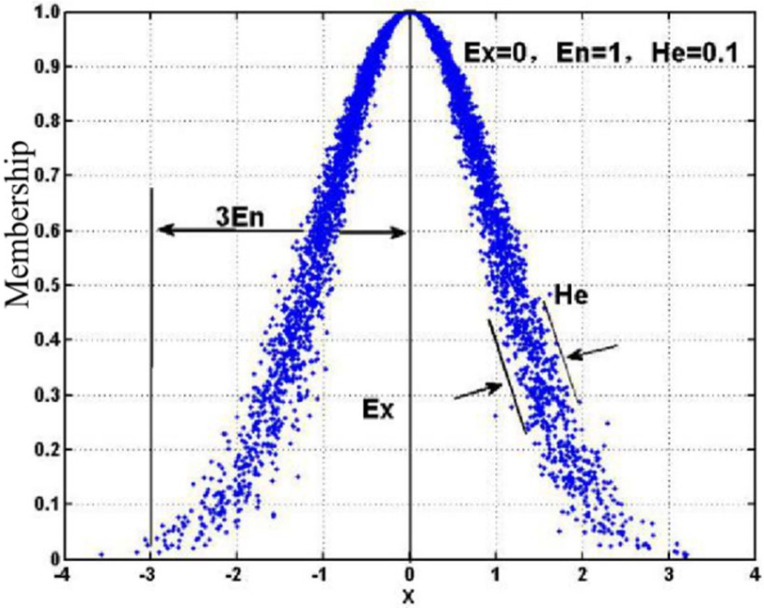
Normal cloud model with parameters.

The cloud model is an evaluation method based on probability statistics and fuzzy set theory [38], and the method mainly characterizes the results through cloud numerical feature parameters $\left(E_{x},E_{n},H_{e}\right)$ [39]. The schematic diagram of the cloud model is shown in Figure 1. When the cloud model approach is used to evaluate the OSHM performance of an enterprise, the main steps are as follows.

**(a) Establish a set of weighting factors for the indicator.** Analytic hierarchy process (AHP) and entropy weight (EW) are two widely used weighting methods. This study uses the combined weight method based on AHP-EW to determine the index weight $W=\left\{\omega_{1},\omega_{2},\cdots ,\omega_{n}\right\}$. The specific formula is as follows [40]:

(1) $$\omega_{j}=-\frac{\mu_{j}\lambda_{j}}{\sum_{1}^{n}\mu_{j}\lambda_{j}}$$

where $\omega_{j}$ is the combined weight of the indicator; $\lambda_{j}$ is the indicator weight value obtained by the AHP method; and $\mu_{j}$ is the weight value obtained by the index EW method.

**(b) Determine the index set and the evaluation domain.** In this study, there are 25 indicators in the enterprise OHSM evaluation index system, which can determine the evaluation index set $\;U=\left\{C_{1},C_{2},\cdots ,C_{n}\right\}$. The evaluation domain is divided into five levels: vigilance level (Level I), improvement level (Level II), transition level (Level III), acceptable level (Level IV), and declarable level (Level V), which can determine the evaluation domain $V=\left\{V_{1},V_{2},\cdots ,V_{m}\right\}$ of each indicator.

**(c) Determine the cloud parameter matrix for each level of each indicator.** It can be calculated by the corresponding cloud parameter formula, and the specific formula is as follows:

(2) $$\{\begin{array}{c} E_{x}=\frac{\left(C_{max}+C_{min}\right)}{2}\\ E_{n}=\frac{\left(C_{max}-C_{min}\right)}{2\sqrt{2\ln2}}\\ H_{e}=k \end{array}$$

where $C_{max}$ is the maximum value of the evaluation index; $C_{min}$ is the minimum value of the evaluation index; and k is a constant, which can be adjusted according to the fuzziness of the comment itself. For the comments of unilateral constraint $C_{max}$ or $C_{min}$, the default expected value can be determined first, and then the cloud parameters can be calculated according to the above formula. Half of the entropy value of the corresponding symmetric cloud can be selected as the entropy value of each cloud, which is described by a half rising and half falling cloud.

**(d) Calculation of affiliation degree.** The affiliation degree of one experiment is obtained using the X-conditional cloud generator algorithm, and then the final affiliation degree is obtained by repeating k times for each sample. The specific formula is as follows:

(3) $$\{\begin{array}{c} \bar{\beta_{ij}}\left(x\right)=\frac{\sum_{i=1}^{k}{\beta_{ij}}^{m}}{k}\\ \overset{=}{\beta_{ij}}\left(x\right)=\frac{\bar{\beta_{ij}}\left(x\right)}{\sum_{j=1}^{n}\bar{\beta_{ij}}\left(x\right)} \end{array}$$

where $\overset{=}{\beta_{ij}}\left(x\right)$ is the final membership of the j indicator of the i sample; $\bar{\beta_{ij}}\left(x\right)$ is the average of the k-degree membership calculations for the ith indicator of the *i* sample value; ${\beta_{ij}}^{m}$ is the degree of membership of the *m* comment of the j indicator of the i sample obtained in one experiment.

**(e) Determine the evaluation level.** Fuzzy transformation is performed on the obtained weight vector and the affiliation degree matrix of each evaluation object, and then the evaluation result is obtained. The specific formula is as follows:

(4) $$\{\begin{array}{c} C=W∴D=\left(b_{1},\;b_{2},\;\cdots b_{m}\right)\\ b_{m}=\overset{n}{\underset{j=1}{\sum }}\omega_{j}\cdot \beta_{ij}{}^{m} \end{array}\;$$

where C is the evaluation result vector; W is the combined-weight vector, and its physical meaning is the importance of each indicator; D is the affiliation matrix formed by $\overset{=}{\beta_{ij}}\left(x\right)$; ∴ is the fuzzy operation; and $b_{m}$ is the affiliation degree of the m comment of the evaluation object.

## 3. Results and Discussion

### 3.1. Overall Status in the OHSML of Listed Companies in China’s Energy Industry

#### 3.1.1. Overall Status Analysis of All Samples

**Figure 2:**
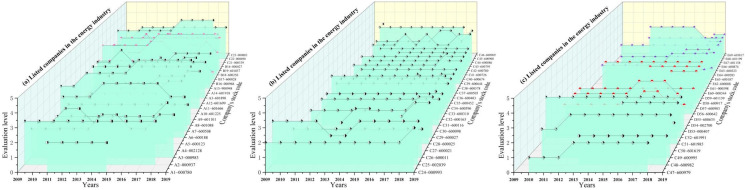
Comprehensive evaluation results of the OHSML of each company from 2009 to 2019. Note: A1–A17 are coal mining, washing, and processing type companies; B18–B23 are oil and gas extraction and processing type companies; C24–C41 are electricity and heat production and supply type companies; D42–D49 are gas production and supply type companies; E80–E99 are water production and supply type companies.

To clarify the OHSM performance of listed companies in China’s energy industry, this study used the cloud model to conduct evaluations of each sample company and visualized the OHSML evaluation results of each company. The results are shown in Figure 2.

Figure 2 shows the evaluation results of the OHSML of 69 listed companies in the energy industry from 2009 to 2019. As shown in Figure 2, among the energy industry companies that have released relevant information (social responsibility report/sustainability report), 92.56% (S = 498) of the sample companies’ OHSM comprehensive evaluation grades fell into the range of “transition level (Level III)” and “improvement level (Level II)”. Only 5.58% (S = 30) of the samples’ OHSM comprehensive evaluation grades belonged to the levels of “acceptable level (Level IV)” and “declarable level (Level V)”. Considering the 72 listed companies that did not disclose any OHSM related information between 2009 and 2019, the percentage of overall energy listed companies with an “Level IV or Level V “was less than 2.25%. This showed that the OHSM performance of most listed companies in the energy industry was still at a low-end level. This could be due to the following two aspects. First, many listed companies have not incorporated occupational health and safety elements into the same important position as economic elements for operation and management [41], which would results in poor performance of these companies in the management framework, management process, and management effectiveness of OHSM. Second, although some companies have implemented OHSM-related measures, many companies do not disclose or partially disclose OSHM-related information [42], which would also results in a lower overall rating. Wang et al. (2021) found that the absence of an authoritative and systematic disclosure framework for listed companies leads some listed companies to choose not to disclose or partially disclose relevant information, which could lead to lower overall evaluation results [37], which is consistent with the findings of this study.

#### 3.1.2. Overall Status Analysis of the Sub-Industry Samples

This study further compared the OHSM performance of the coal mining, washing, and processing industry, oil and natural gas extraction and processing industry, electricity and heat production and supply industry, gas production and supply industry, and water production and supply industry. From the perspective of the average level, there were differences in the OHSM level of different industries. During the study period, the average levels of OHSM of enterprises in different industries were as follows: coal mining, washing, and processing industry > petroleum and natural gas extraction and processing industry > gas production and supply industry > electricity power and heating production and supply industry > water production and supply industry. The average value of OHSM level of coal mining, washing, and processing industry was the highest, reaching the level of “Level III ~ Transition Level”, and the average level of other sub-sectors was lower than this level. Wang et al. (2021) found that coal industry enterprises have a harsh operating environment, frequent safety accidents and occupational diseases in their production operations, and the government and other relevant departments have stricter constraints on the safety management of this type of enterprises [43]. At the same time, actively improving the OSHM level is important to protect the health of employees and promote the sustainable development of enterprises, and coal mining enterprises subjectively need to actively improve their performance in terms of OHS [6,44]. It is possible that these reasons have led coal industry companies to pay more attention to OHSM, and thus the level of OHSM performance of coal industry companies is relatively high.

Further research found that the 2019 OHSM benchmarking enterprises in the coal mining and washing industry, oil and natural gas extraction industry, electric heating power production and supply industry, gas production and supply industry, and water production and supply industry are China Shenhua (V), Sinopec (IV), China Nuclear Power (III), Shenzhen Gas (III), Hanlan Environment (III), etc. These enterprises are the guiders and leaders of OHSM reform and development, and their relevant management experience can guide other companies.

### 3.2. Change Trend Analysis in the OHSML of Listed Companies in China’s Energy Industry

#### 3.2.1. Change Trend Analysis at the Overall Level

**Figure 3:**
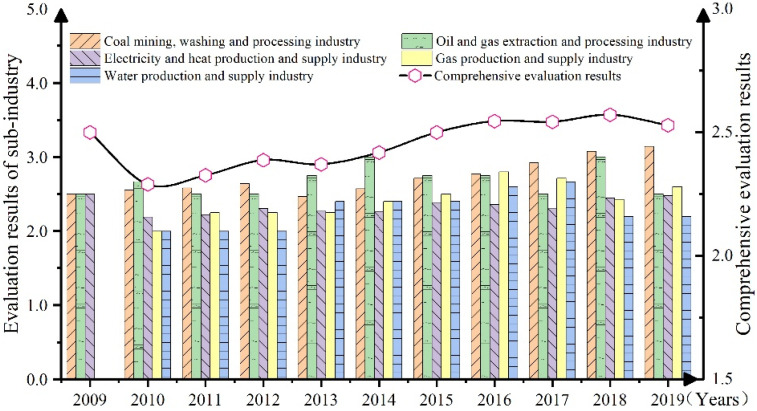
Overall and sub-industry OHSML evaluation results from 2009 to 2019.

To clarify the dynamic changes of the OHSM performance of listed companies in the energy industry, this study further analyzed the dynamic changes of the overall OHSM performance of the sample companies from 2009 to 2019 (Figure 3).

Figure 3 shows the OHSML evaluation results of the overall and sub-industry from 2009 to 2019. As can be seen from Figure 3, on the whole, although the results of the annual OHSM comprehensive evaluation of listed companies in China’s energy industry from 2009 to 2019 showed an upward trend, the growth rate was low, and the overall level still did not reach “Level IV~acceptable level” grade. This showed that the OHSML of listed companies in China’s energy industry is constantly improving. This is mainly due to the fact that between 2006 and 2019, China carried out a series of social responsibility advocacy activities from top to bottom and issued a series of guidelines and standards to urge relevant companies to take active measures in social and environmental areas, including safety and health management [45]. At the same time, some companies have gradually realized the importance of OHSM and have improved in OHSM and other aspects. However, the growth rate of OHSML of listed companies in China’s energy industry is relatively low, and the overall level has not yet reached the “transition level”. This is mainly due to the fact that although some companies have implemented some OHS management projects, the implementation of environment management work has increased the economic burden and management difficulties of enterprises to a certain extent [37], so many companies could have limited implementation and limited investment in OHSM, which has led to a relatively low growth rate of OHS management in these companies, and the overall level has not yet reached the “transition level”.

#### 3.2.2. Change Trend Analysis at the Sub-Industry Level

This study further compared the annual changes in OHSM performance in the coal mining, washing, and processing industry, oil and natural gas extraction and processing industry, electricity and heat production and supply industry, gas production and supply industry, and water production and supply industry (Figure 3). From the perspective of annual changes, the OHSML of various industries has shown a trend of rising volatility, but the growth rate is extremely low, and the changes in OHSML of different industries are different. During the study period, the growth rate of OHSM level of different industries in descending order is gas production and supply industry > coal mining and washing and processing industry > oil and natural gas extraction and processing industry > water production and supply industry > electricity and heat production and supply industry. This indicates that the energy industry enterprises have slightly improved OHSM during 2009–2019. This may be because enterprises pay less attention to OHSM and disclose less [42], so the improvement of OHSM level is not apparent. This suggests that relevant government departments should strengthen the supervision and evaluation of OHSM of enterprises and guide the OHSM of enterprises in different industries in a targeted manner to encourage enterprises to continuously improve their OHSM level.

This study further sorted out and analyzed the OHSML comprehensive evaluation results of sample companies in each sub-industry during 2009–2019. According to the changing trend of the OHSM level of each company, listed companies in the energy industry can be roughly divided into three types: companies with rising volatility, companies with continuous stability, and companies with falling volatility. Among them, the typical sample companies with rising volatility characteristics include China Shenhua (III→V), Sinopec (III→IV), Mindong Electric Power (II→III), and Shenzhen Gas (III→IV). The typical sample companies with continuous stability characteristics include Lu’an Environmental Energy (III→III), Guanghui Energy (III→III), Shenzhen Energy (III→III), and Chongqing Gas (III→III). The typical sample companies with falling volatility characteristics include Shanghai Energy (III→II), Blue Flame Holdings (III→II), Huadian Energy (III→II), and Xinjiang Haoyuan (III→II). To further improve the overall OHSM level of listed companies in the energy industry, it is necessary to conduct an in-depth analysis of these three types of typical sample companies to clarify the specific reasons for the changes, especially companies with declining volatility characteristics, in order to provide guidance for further improvement of the OHSM level.

### 3.3. Characteristics Analysis in the OHSML of Listed Companies in China’s Energy Industry

#### 3.3.1. Characteristics Analysis at the Subsystem Level

**Figure 4:**
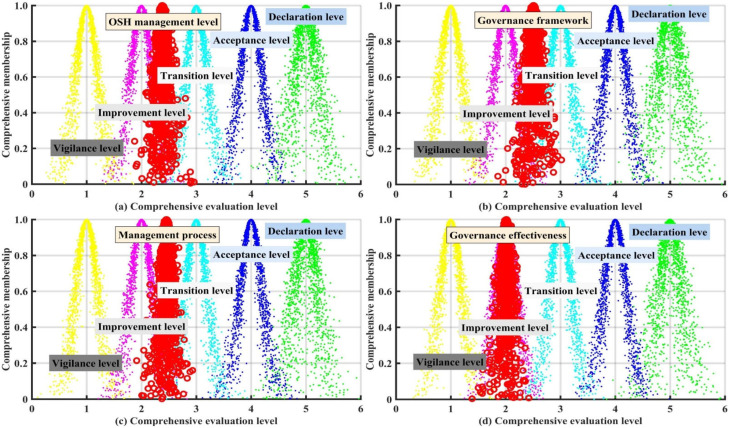
Comprehensive evaluation results of the overall and FPE three-dimensional subsystem.

To clarify the weak links of the OHSM of listed companies in the energy industry, this study further analyzed the OHSM performance of the companies from the three-dimensional subsystem level of “management framework, management process, management effectiveness” (FPE). The results are shown in Figure 4.

Figure 4 showed the OHSML evaluation results of the overall and FPE three-dimensional subsystems of listed companies in the energy industry. As shown in Figure 4a, the overall OHSM cloud expectation $E_{x}=2.3991$ of the listed companies in the energy industry falls between the “improvement level” and the “transition level” and is more biased towards the “improvement level” evaluation cloud, which indicates that the OHSM level of the listed companies in the energy industry is between the “improvement level” and the “transition level”. From the perspective of the PFT three-dimensional subsystems of OHSM of listed companies in the energy industry (Figure 4b–d), the evaluation level of the governance framework subsystem is the highest ($E_{x}$ = 2.5474, transition level), whereas the evaluation level of the management process subsystem ($E_{x}$ = 2.4474, improvement level) and management effectiveness subsystem ($E_{x}$ = 2.0778, improvement level) are relatively low. The evaluation level of the governance framework system is higher than that of the management process subsystem and the management effectiveness subsystem. The possible reason for this is that at this stage some legal policies clearly specify the requirements of companies in terms of OHS management framework and other aspects [45]. In addition, many companies are gradually recognizing the importance of actively improving their OSHM performance for sustainable development [6], and these companies are proactively improving their OHS management top-level design, which could results in relatively high scores for the governance framework subsystem. However, the expectation of the management effectiveness subsystem is lower than the expectation of the management process subsystem. The possible reason is that the measurement index of the management efficiency subsystem of enterprise occupational safety and health is mainly a quantitative index, such as per capita safety and health input, the accident death rate per 1000 people, the new incidence of occupational diseases per 1000 people, etc. The improvement of enterprises in these aspects is often accompanied by the increase of management investment, the innovation of related equipment and technology, and the increase of management costs [37]. This makes it difficult for companies to improve objectively in these areas. In general, the FPE three-dimensional subsystems of OHSM of listed companies in the energy industry are still at the low level, especially in the aspects of OHS management process subsystem and OHS management effectiveness subsystem.

#### 3.3.2. Characteristics Analysis at the Element Level

**Figure 5:**
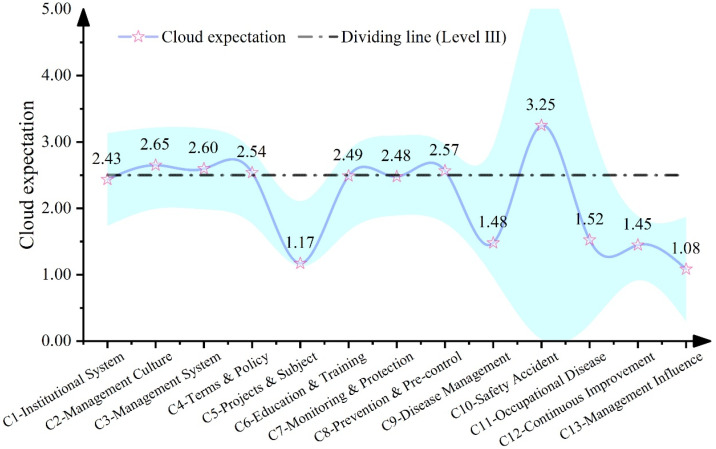
Expected value distribution of the cloud model for element layer indicators.

To further clarify the weak links in the OHSM of listed companies in the energy industry, this study further visualized the OHSML cloud expected value of the element layer. The results are shown in Figure 5.

Figure 5 shows the OHSML evaluation results of the element layer of listed companies in the energy industry. As can be seen from Figure 5, the expected value of most element layer indicators fluctuates between the improvement level (Level II) and transition level (Level III), which indicates that the listed companies in the energy industry are relatively weak in all elements of OHSM at the present stage. This reminds us that currently listed companies in the energy industry need to pay more attention to OHSM. OHS management culture is very important to corporate safety and health management; it can lead the way in corporate safety and health management practices, and when the management culture does not lead, then the organization cannot change quickly [46]. This study found that the safety and OHS management culture of listed companies in the energy industry is not at a leading (level 4 or 5) level, which would prevent the rest of the requisite changes from occurring. Further analysis of OSHM weak links shows that most of the sample companies performed relatively well in management culture (C2), management system (C3), terms and policies (C4), prevention and pre-control (C8), safety accidents (C10), etc. The comprehensive evaluation results of these indicators have reached “transition level” and above. However, the performance is relatively weak in terms of project topics (B5), detection and protection (C7), disease management (C9), continuous improvement (C12), and management impact (B13). The comprehensive evaluation results of these indicators have not reached “transition level” and above. The comprehensive evaluation results of a few element layer indicators, such as management system (C3), terms and policies (C4), prevention and pre-control (C8), and safety accidents (C10), are relatively high. This is mainly because some laws and regulations clearly stipulate that listed companies in the energy industry need to pass OHSM certification, etc. Therefore, some listed companies in the energy industry perform better in this regard, so the scores of these indicators are relatively high. However, the comprehensive evaluation results of element layer indicators such as project and subject (C5), disease management (C5), continuous improvement (C12), and management influence (C13) are relatively low. This is mainly due to the fact that improvements in such indicators are often accompanied by innovations in related equipment technology, increases in management costs, etc., which could lead to a low incentive for companies to improve in these areas, less disclosure of quantitative information [47], and thus a relatively low overall construction performance. The low level of OHS management culture, OHS management process, and OHS management performance would restrict the progress of achieving the national strategic goal of “Healthy China”. At the same time, these weak links are the key to further improve the OHSM level of listed companies in the energy industry.

## 4. Conclusions and Countermeasures

### 4.1. Conclusions

Based on in-depth thinking about the existing OHSM evaluation, this research incorporated “management framework, management process, management effectiveness” (FPE) into the integrated framework and constructed the FPE evaluation system of enterprise OHSM. We also innovatively collected and refined FPE information from the perspective of information disclosure, and used the combined weight cloud model to evaluate and analyze the OHSML of listed companies in China’s energy industry.

(1) The overall status analysis results showed that the OHSML of most listed companies in China’s energy industry was still at a low-end level. Among the companies that have issued relevant information reports, only 5.58% (S = 30) of the sample companies’ OHSML were at acceptable level (Level IV) or declarable level (Level V). The OHSML comprehensive evaluation level of 92.56% (S = 498) of the sample companies was between the transitional level (Level III) and the improved level (Level II). This indicates that the performance of listed companies in the energy industry in OHSM is not optimistic.

(2) The dynamic change analysis results showed that during 2009–2019, although the annual OHSML of listed companies in China’s energy industry showed an upward trend, the growth rate was low, and even the OHSML of some listed companies in the energy industry showed the characteristics of reduced fluctuations. This indicates that the improvement in OHSM of listed companies in the energy industry during 2009–2019 was relatively small.

(3) The characteristics analysis results showed that, from the perspective of the PFT three-dimensional subsystem level of OHSM, the evaluation level of the governance framework subsystem was the highest, whereas the evaluation level of the management process subsystem and the management effectiveness subsystem were relatively low. Specifically, most listed companies in the energy industry had weak performance in terms of project and subject (C5), disease management (C5), continuous improvement (C12), and management influence (C13), as well as other indicators. These weak links are the key for listed companies in the energy industry to further improve their OHSM level.

### 4.2. Suggestions

Based on the above research findings, this study proposes the following strategies to improve the OHSM performance of listed companies in the energy industry.

(1) In the process of information collection and refining, this study found that the degree of standardization of OHSM-related information disclosure is not high. For example, some listed companies in the energy industry do not disclose their OHSM-related information, and some listed companies in the energy industry selectively disclose a small amount of OHSM-related information. In general, the quality of OHSM-related information disclosed by listed companies in the energy industry is poor, which leads to relatively low evaluation results for some companies. Based on this, we recommend that the government and other relevant administrative departments should strengthen the regulation and supervision of OHSM information disclosure. Specifically, the OHSM-related information disclosure framework system can be constructed to clarify the hotspots of enterprise-related information disclosure; the minimum disclosure standards for OHSM-related information can be set to standardize the relevant information disclosure format; and the OHSM-related information authentication rating can be introduced to strengthen the supervision of enterprise-related information disclosure.

(2) The research results show that the OHSML of most of the listed companies in the energy industry is still at the low level, and its growth rate is relatively low, and even the OHSM level of some of the listed companies in the energy industry shows the characteristics of weakening fluctuations. This indicates that the listed companies in the energy industry have negligible improvement in OHSM from 2009 to 2019. Based on this, we suggest that the government and other relevant management departments should actively organize and carry out OHSM evaluation work and guide enterprises to make improvements in OHSM. Specifically, an OHSM management evaluation system (including a data collection management platform) should be established to keep abreast of the changing trends of the OHSM level of each company and to implement standardized and precise accountability for companies that show the characteristics of weakening volatility; OHSM evaluation projects should be released regularly to guide relevant subjects such as universities and research institutions to actively participate in the process of corporate OHSM improvement. The OHSM model enterprise selection activities should be carried out, supplemented by rewards and punishments, to guide relevant enterprises to maintain and influence and drive other enterprises to improve their OHSML.

(3) The results show that in the FPE subsystem of OHSM, the evaluation level of the management process subsystem and management effectiveness subsystem are relatively low. Specifically, the OHSM of listed companies in the energy industry has poor performance in project topics, disease management, continuous improvement, and management impact. These weak links are the direction for listed companies to further improve OHSML in the future. Based on this, we suggest that the government and other relevant administrative departments should focus on guiding enterprises to improve the weak links. Specifically, the OHSM weak link exchange activities can be carried out to publicize the advanced management experience and management methods of outstanding companies in this area. Policies such as health credit, health securities, and health investment tax incentives can be used to reduce the cost of companies in improving this aspect and guide listed companies in the energy industry to improve the key weaknesses of OHSM.

## Figures and Tables

**Table 1:** Enterprise occupational health and safety management evaluation index system.

Target Layer	Subsystem Layer	Elements Layer	Basic Index Layer
A—Enterprise Occupational Health and Safety Management	B1—Management Framework	C1—Institutional System	X1: The degree of completeness of the management system, such as whether there is a safety and health management system, occupational disease prevention and control management methods and other regulations (1–5 points) X2: The degree of completeness of department settings, such as whether there are permanent institutions such as safety management agencies, occupational health agencies, employee rights protection agencies, and safety and health committees (1–5 points)
		C2—Management Culture	X3: The organization’s emphasis on OHS management, such as the organization’s vision, mission, values, safety and health topics, including safety and health statements (1–5 points) X4: The degree of enrichment of the implementation of related cultural activities, such as whether to actively carry out OHS related cultural activities such as competitions, presentations, signatures, and essays (1–5 points)
		C3—Management System	X5: The completeness of the relevant management system, such as whether it has passed the occupational health and safety management system certification, and contains a series of OHS management systems such as safety management, occupational disease prevention, and employee file insurance (1–5 points) X6: The degree of systematicness of the relevant management system, if it contains descriptions of OHS-related management principles, management systems, management standards, etc. (1–5 points)
		C4—Terms and Policies	X7: The degree of standardization of compliance with relevant laws and regulations, such as whether they strictly follow the “Labor Contract Law”, “Occupational Disease Prevention Law”, and other laws and regulations (1–5 points) X8: The degree of completeness of OHS clauses in relevant laws, such as whether suppliers are required to provide OHS system certification, certification of compliance, evaluation by external experts, etc. (1–5 points)
	B2—Management Process	C5—Project and Subject	X9: The degree of participation in OHS-related courses of the company, such as whether to undertake or participate in OHS-related domestic and foreign innovation topics/strategic topics/industry-standard formulation, etc. (1–5 points)
		C6—Education and Training	X10: The degree of enrichment of relevant education and training, such as whether a series of training and education activities such as on-site teaching, online learning, and special training is carried out X11: Coverage of relevant education and training (per capita training time)
		C7—Monitoring and Protection	X12: The completeness of employee personal protection, such as whether employees are equipped with advanced and effective protective equipment, professional medical equipment, rescue facilities, etc., whether OHS related insurance and physical examinations are implemented (1–5 points) X13: The degree of importance the organization attaches to employee mental health management, such as whether a series of measures such as mental health consultation room construction, psychological consultation training, mental health promotion, etc., have been taken (1–5 points)
		C8—Prevention and Pre-control	X14: The degree of standardization of operating environment management, such as whether to implement measures such as regular control, inspection and evaluation of dust, noise, toxic substances, etc. (1–5 points) X15: The completeness of the implementation of emergency support management, such as whether emergency support measures, capital investment, professional equipment, professionals, etc. are complete X16: The completeness of the implementation of hidden danger investigation and management, such as whether special inspections, expert consultations, rectification assessments, and other measures are actively carried out (1–5 points)
		C9—Disease Management	X17: The degree of completeness of occupational disease prevention, such as whether a series of prevention and control measures such as the construction of prevention and control work system, equipment research and development updates, personal protection, publicity, and education have been carried out (1–5 points) X18: The completeness of the on-the-job management of the sick employee, such as whether the sick employee has proper rehabilitation treatment and job transfer placement, etc. (1–5 points)
	B3—Management Effectiveness	C10—Safety Incident	X19: The severity of relevant accidents, that is, the death rate per thousand accidents (%) X20: The frequency of related accidents, that is, the accident rate per million working hours (%)
		C11—Occupational Disease	X21: Severity of related occupational diseases, that is, the new incidence rate of occupational diseases per thousand people (%)
		C12—Continuous Improvement	X22: The degree of improvement of related safety incidents, that is, the reduction rate of safety incidents (%) X23: The degree of improvement in the incidence of related occupational diseases, that is, the reduction rate of new occupational diseases (%) X24: The degree of improvement of related investment, that is, the growth rate of OHS capital investment (%)
		C13—Management Impact	X25: The influence of related management practices, such as whether there are OHSM-related awards/honours/patents/papers, etc. (1–5 points)

## Data Availability

The data that support the findings of this study are available from the corresponding author upon reasonable request.
